# Impact of the COVID-19 Pandemic and Obstetrician and Gynecologist Workforce Distribution on Vaccine Deployment and Predicting Women’s Healthcare Shortages

**DOI:** 10.7759/cureus.14545

**Published:** 2021-04-18

**Authors:** Toral Shastri, Chandruganesh Rasendran, Metabel Markwei, Christine Hur, Oluwatosin Goje

**Affiliations:** 1 Obstetrics and Gynecology, Midwestern University Chicago College of Osteopathic Medicine, Downers Grove, USA; 2 Department of Ophthalmology, Case Western Reserve University School of Medicine, Cleveland, USA; 3 Department of Obstetrics and Gynecology, Cleveland Clinic Lerner College of Medicine, Cleveland, USA; 4 Obstetrics and Gynecology & Women’s Health Institute, Cleveland Clinic, Cleveland, USA

**Keywords:** covid-19, women’s health, obstetricians and gynecologists, coronavirus pandemic

## Abstract

Background

Obstetricians and gynecologists, who serve a vital role in providing women’s healthcare in the United States, are at risk of COVID-19 exposure via asymptomatic patients and deliveries. This study analyzes state-level geographical distribution of COVID-19 cases and age distribution of Obstetricians and gynecologists (OB/GYNs) to project which US regions will experience a more significant COVID-19 patient burden and provides a guide for vaccine distribution in the OB/GYN workforce.

Methods

The Association of American Medical Colleges' state-level workforce data is combined with COVID-19 case data reported by Johns Hopkins University. All data and code are available at https://github.com/cxr244/covid-obgyn.

Results

Our findings illustrate that OB/GYNs in the Midwestern region of the US experience the highest number of COVID-19 patients per OB/GYN over 60 years of age: North Dakota, South Dakota, Iowa, Wisconsin, and Idaho have the highest burden of COVID-19 patients per OB/GYN, warranting vaccine distribution priority. Additionally, states with the highest proportion of OB/GYNs over the age of 60 like Florida (38%), New Mexico (37%), Alabama (36%), California (36%), and New Jersey (34%), should be strongly considered for priority vaccine allocation, to mitigate predicted OB/GYN workforce shortages.

Conclusion

When planning and executing vaccine allocation, especially in the early stages of distribution, it is critical to evaluate which communities can benefit the greatest from the limited number of vaccines. A strategy of distribution of COVID-19 vaccines to older physicians with a more significant COVID-19 burden can minimize shortages of providers within these states and ensure adequate access to women’s healthcare within the communities they serve.

## Introduction

The COVID-19 pandemic is rapidly spreading across the United States and is disproportionately affecting individuals over the age of 60 [1]. Obstetricians and gynecologists (OB/GYNs) serve a vital role in providing women’s healthcare in the United States and are at risk via exposure to asymptomatic patients and surgical procedures [2,3]. The high risk of COVID-19 infection may lead to OB/GYNs, particularly those over the age of 60, to discontinue the practice, work part-time, or practice virtually at an increasing rate. While the future impact of COVID-19 is unknown, this loss of providers is likely to exacerbate the pre-existing barriers women face in access to healthcare [4]. Vaccinating against COVID-19 has proved to be a successful countermeasure to COVID-19 exposure and provides valuable protection for healthcare providers and dissemination to the public [5]. However, with the limit of resources, allocating and administering COVID-19 vaccinations to areas of need require thorough planning and can lessen barriers to women’s healthcare [5]. To vaccinate an estimated 100 million people by April 2021 [6], the judicious allocation of limited vaccines to areas burdened by the highest COVID-19 cases per provider ratio will accelerate efforts to quell the burgeoning pandemic. This study overlays the geographical prevalence of COVID-19 cases with the age distribution of OB/GYNs at the county level to project which US regions are at increased risk of experiencing severe OB/GYN shortages soon. These results serve as a potential guide for vaccination distribution to the OB/GYN workforce. This article was previously presented as a research poster at the 2020 American Medical Association annual research symposium from December 3, 2020 to December 6, 2020.

## Materials and methods

State-level workforce data reported by the Association of American Medical Colleges is combined with COVID-19 case data reported by Johns Hopkins University [7,8]. Workforce data is standardized by the population of women from the US Census Bureau for each state as a proxy for the number of potential patients requiring care by OB/GYNs [9]. State population data is collected from the 2018 National and State Population Estimates of the US Census Bureau [10]. COVID-19 vaccination data is collected from Bloomberg’s COVID-19 vaccine tracker for the general population [11]. It was not possible to acquire vaccination data for OB/GYNs at this time. Cross-sectional data analysis and visualization are done using the tidyverse and maps packages in the R programming language [10,12,13]. All data and code are available at https://github.com/cxr244/covid-obgyn. Since all data is publicly available without Protected Health Information, this study is IRB exempt.

## Results

**Figure 1:**
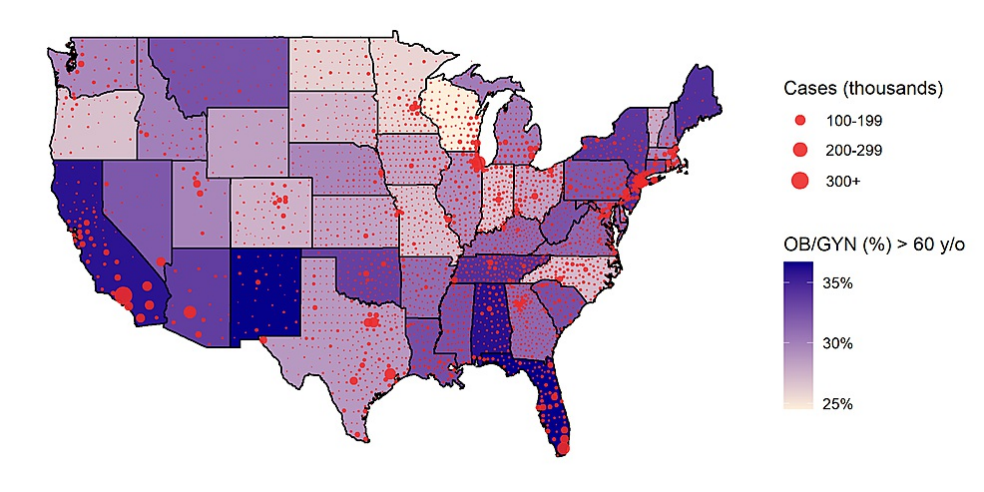
Choropleth of percent OB/GYNs over 60 years of age and COVID-19 infections at the county level. State shade represents the percentage of practicing OB/GYNs over 60 years of age, with darker shades indicating a higher percentage of practicing OB/GYNs over 60 years old [7]. Red bubbles represent the number of confirmed COVID-19 cases per county as of December 21, 2020 [8]. The radii of the bubbles are proportional to the number of confirmed COVID-19 cases within the corresponding county. OB/GYNs: Obstetricians and gynecologists.

As of December 21, 2020, there were nearly 18 million COVID-19 cases across the United States. The heterogeneous distribution of COVID-19 cases in the United States overlaid with the proportion of practicing OB/GYNs over 60 years of age further highlights hotspots for future OB/GYN shortages due to more severe disease complications in the elderly (Figure 1). A total of 31% of practicing OB/GYNs in the US are over 60 years old. The states with the highest proportion of OB/GYNs over 60 years of age are Florida (38%), New Mexico (37%), Alabama (36%), California (36%), and New Jersey (34%). Worsening shortages in the OB/GYN workforce are predicted in the Midwestern region of the United States because these states have the highest number of female COVID-19 patients per OB/GYN over 60 years of age (North Dakota, South Dakota, Iowa, and Wisconsin) (Table 1).

**Table 1:** States with the highest burden of COVID-19 cases per OB/GYN over 60 years of age. Exposure risk was quantified as the number of confirmed COVID-19 cases in women as of December 21, 2020, per OB/GYN over age 60 [7,8]. The five US states with the highest exposure risk and percentage of population vaccinated (as of December 26, 2020) are listed; the entire list can be found at the reproducible repository (see Methods) [11]. OB/GYNs: Obstetricians and gynecologists.

State	# OB/GYNs over 60 years of age (% of total OB/GYNs)	Total female patient burden per OB/GYN over 60 years of age	COVID-19 female patient burden per OB/GYN over 60 years of age	% of population vaccinated
North Dakota	19 (26%)	5,052	2,301	1.7%
South Dakota	26 (27.4%)	4,600	1,811	1.5%
Iowa	79 (28.2%)	5,684	1,715	0.27%
Wisconsin	152 (24.5%)	4,714	1,635	0.18%
Idaho	49 (30.1%)	5,349	1,328	0.57%

The states with the lowest proportion of OB/GYNs over 60 years old are Wisconsin (25%), Minnesota (26%), North Dakota (26%), Indiana (27%), and Oregon (27%). There are 114 COVID-19 patients per OB/GYN over 60 years old in Vermont, making it the lowest number of COVID-19 patients per OB/GYN (Table 2). The states with the lowest female patient burden per OB/GYN over 60 years of age are Rhode Island (2,864), Maryland (2,903), Connecticut (2,922), Hawaii (3,008), and New York (3,054).

**Table 2:** States with the lowest burden of COVID-19 cases per OB/GYN over 60 years of age. Exposure risk was quantified as the number of confirmed COVID-19 cases in women as of December 21, 2020, per OB/GYN over age 60 [7,8]. The five US states with the lowest exposure risk and percentage of population vaccinated (as of December 26, 2020) are listed; the entire list can be found at the reproducible repository (see Methods) [11]. OB/GYNs: Obstetricians and gynecologists.

State	# OB/GYNs over 60 years of age (% of total OB/GYNs)	Total female patient burden per OB/GYN over 60 years of age	COVID-19 female patient burden per OB/GYN over 60 years of age	% of population vaccinated
Vermont	29 (27.9%)	3,060	114	0.7%
Hawaii	73 (30.9%)	3,008	142	Not available
Maine	57 (34.3%)	4,111	173	1.3%
Oregon	159 (26.8%)	3,560	329	0.34%
New Hampshire	55 (30.4%)	3,797	344	0.28%

**Figure 2:**
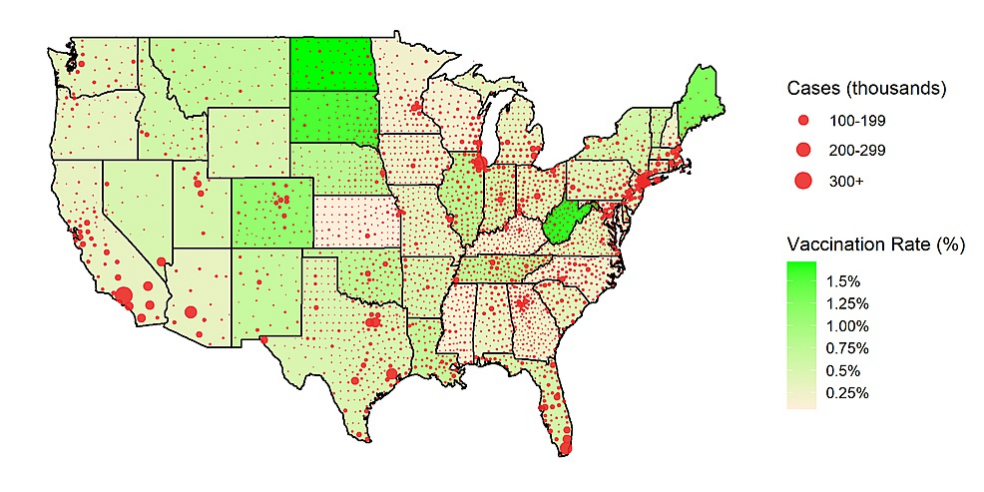
Choropleth of COVID-19 vaccination rate and COVID-19 infections at the county level. State shade represents the percentage of the population administered a COVID-19 vaccine, with darker shades indicating a higher vaccination rate [11]. Red bubbles represent the number of confirmed COVID-19 cases per county as of December 21, 2020 [8]. The radii of the bubbles are proportional to the number of confirmed COVID-19 cases within the corresponding county [8].

On December 14, 2020, the first COVID-19 vaccine was given in the US, beginning its most extensive immunization campaign [11]. According to the United States Centers for Disease Control and Prevention (CDC), 1.64 million doses of the COVID-19 vaccine had been administered as of December 26, 2020 [11]. North Dakota (1.7%), Alaska (1.6%), West Virginia (1.6%), South Dakota (1.5%), and Colorado (1.1%) were the states with the highest percentage of their population currently vaccinated (Figure 2) [11]. Our findings illustrate that states like South Dakota, Iowa, Wisconsin, and Idaho, with the highest number of COVID-19 patients per OB/GYN over 60 years old, warrant vaccine distribution priority. While North and South Dakota currently record higher numbers of vaccine administration, our data argue for greater attention for some of the other states with similarly high COVID-19 patients to OB/GYNs over the age of 60 (e.g., Iowa and Wisconsin). Additionally, states with the highest proportion of OB/GYNs over the age of 60 like Florida (38%), New Mexico (37%), Alabama (36%), California (36%), and New Jersey (34%), should be strongly considered for priority vaccine allocation, to mitigate predicted OBGYN workforce shortages [14].

## Discussion

The high prevalence of COVID-19 in the United States is attributed to an asymptomatic incubation period with or without detectable virus and ease of transmission [15,16]. This characteristic significantly affects healthcare personnel directly or indirectly exposed to patients or infectious materials [17]. 

OB/GYNs over 60 years old practicing in North Dakota, South Dakota, Iowa, Wisconsin, and Idaho have the highest exposure to COVID-19 patients and may be at increased risk for an OB/GYN shortage in the near future due to the COVID-19 pandemic. Compared to other medical specialties, OB/GYNs face an increased risk of COVID-19 infection due to their constant exposure to predominantly young women of childbearing age and mild or asymptomatic pregnant patients [2,3].

The practicing OB/GYN’s average age is currently 51 years old [18]. With a predicted shortage of 9,000 OB/GYNs across the United States by 2020, an early retirement wave is likely to exacerbate the current lack of physicians for women’s healthcare [14]. OB/GYNs facing the highest COVID-19 patient burden are clustered in the Midwestern region: a region with existing barriers to women’s care within rural counties [19]. An increase in the shortage of OB/GYN providers within this region can decrease accessibility to women’s care and lead to worse outcomes in prenatal, perinatal, postpartum, and preventative care [20]. Also, post-menopausal women, who are at greater risk of cardiovascular disease, breast cancer, and osteoporosis may experience lower access to care [21].

## Conclusions

When planning and executing vaccine allocation, especially in the early stages of distribution, it is critical to evaluate which communities can benefit the greatest from the limited number of vaccines. Older physicians practicing in North Dakota, South Dakota, and Iowa experience the highest burden of COVID-19 patients compared to all other states. OB/GYNs in these states have a 15 times greater COVID-19 caseload than states with the lowest COVID-19 case burden per OB/GYN over 60 years of age, such as Vermont, Hawaii, and Maine. A strategy of distribution of COVID-19 vaccines to older physicians with a more significant COVID-19 burden can minimize shortages of providers within these states and ensure adequate access to women’s healthcare within the communities they serve.
